# The Role of the Human Entorhinal Cortex in a Representational Account of Memory

**DOI:** 10.3389/fnhum.2015.00628

**Published:** 2015-11-20

**Authors:** Heidrun Schultz, Tobias Sommer, Jan Peters

**Affiliations:** ^1^Department of Systems Neuroscience, University Medical Center Hamburg-EppendorfHamburg, Germany; ^2^Department of Education and Psychology, Freie Universität BerlinBerlin, Germany

**Keywords:** entorhinal cortex, medial temporal lobe, memory, fMRI, hippocampus, perirhinal cortex, parahippocampal cortex

## Abstract

Connectivity studies in animals form the basis for a representational view of medial temporal lobe (MTL) subregions. In this view, distinct subfields of the entorhinal cortex (EC) relay object-related and spatial information from the perirhinal and parahippocampal cortices (PRC, PHC) to the hippocampus (HC). Relatively recent advances in functional magnetic resonance imaging (fMRI) methodology allow examining properties of human EC subregions directly. Antero-lateral and posterior-medial EC subfields show remarkable consistency to their putative rodent and nonhuman primate homologs with regard to intra- and extra-MTL functional connectivity. Accordingly, there is now evidence for a dissociation of object-related vs. spatial processing in human EC subfields. Here, variance in localization may be integrated in the antero-lateral vs. posterior-medial distinction, but may additionally reflect process differences. Functional results in rodents further suggest material-specific representations may be more integrated in EC compared to PRC/PHC. In humans, however, evidence for such a dissociation between EC and PRC/PHC is lacking. Future research may elucidate on the unique contributions of human EC to memory, especially in light of its high degree of intrinsic and extrinsic connectivity. A thorough characterization of EC subfield function may not only advance our understanding of human memory, but also have important clinical implications.

## Introduction

A small structure embedded in the anterior temporal lobe (Pruessner et al., 2002), the entorhinal cortex (EC) has garnered attention beyond the neuroscientific community through the 2014 Nobel Prize in Physiology or Medicine to John O’Keefe, May-Britt Moser and Edvard I. Moser. Their discoveries of spatially sensitive cells in the hippocampus (HC) and neighboring EC have gone a long way towards elucidating how animals navigate in space (O’Keefe and Dostrovsky, 1971; Fyhn et al., 2004; Hafting et al., 2005). Beyond navigation, EC, together with the adjacent perirhinal cortex (PRC), parahippocampal cortex (PHC) and HC, forms the medial temporal lobe (MTL) system, considered pivotal to memory (Squire and Zola-Morgan, 1991; Eichenbaum et al., 2007). A thorough body of connectivity studies in animals has identified EC as a relay station within MTL, passing information between HC and neocortex (Lavenex and Amaral, 2000; van Strien et al., 2009). Although closely tied to its role in navigation (Buzsáki and Moser, 2013; Moser et al., 2015), EC’s role in memory has often been investigated in a separate line of research.

Studies in humans underline its importance for memory: EC may be affected early in Alzheimer’s disease (AD) and mild cognitive impairment (MCI; deToledo-Morrell et al., 2004; Pihlajamäki et al., 2009; Markesbery, 2010; Khan et al., 2014). Its volume correlates with memory performance in healthy participants as well as MCI and AD patients (Di Paola et al., 2007; Goto et al., 2011; Fujishima et al., 2014). Functional magnetic resonance imaging (fMRI) studies showed EC engagement during a range of memory functions (Kirwan and Stark, 2004; Bellgowan et al., 2009; Doeller et al., 2010; de Vanssay-Maigne et al., 2011; Hargreaves et al., 2012; Okada et al., 2012; Newmark et al., 2013; Staresina et al., 2013a; Maass et al., 2014; Schon et al., 2015). Finally, deep-brain stimulation of EC during spatial learning improved memory (Suthana et al., 2012). A thorough characterization of human EC, both in terms of connectivity and function, is therefore indispensable to our understanding of the neural basis of memory and its disorders.

Importantly, EC is not a homogeneous region. In animals as in humans, EC contains a number of cytoarchitectonically defined subfields (Amaral et al., 1987; Insausti et al., 1995, 1997; Krimer et al., 1997). MTL connectivity models typically consider two major EC subregions, characterized by distinct anatomical connectivity patterns in rodents and nonhuman primates (Lavenex and Amaral, 2000; van Strien et al., 2009). The function of these subfields may be partially determined by their connectivity. However, relating animal findings to human EC has been difficult: Until recently, fMRI as the major noninvasive imaging technique has had limited success delineating human EC subregions. Precise localization at standard resolution is difficult due to EC’s small size, and the region is sensitive to signal dropout (Carr et al., 2010). Using high-resolution and ultra-high field fMRI, recent studies have yielded valuable information on human EC subfield connectivity and function. In this Mini Review, we review these findings and discuss how they relate to the animal literature.

## Connectivity and Functional Implications

**Figure 1:**
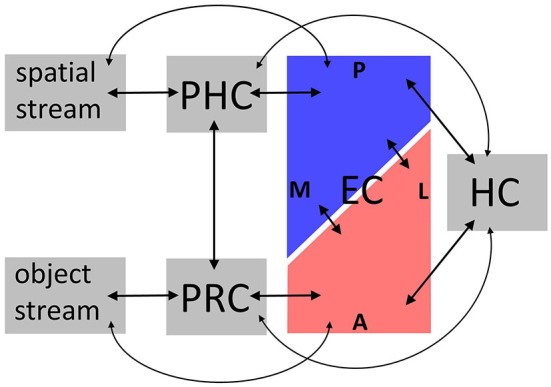
**Overview over connectivity findings.** Anatomical connectivity studies in animals and functional connectivity studies in humans converge on the following: bilateral connections convey spatial information between dorsal visual regions, parahippocampal cortex (PHC), posterior-medial entorhinal cortex (EC), and hippocampus (HC), and object information between ventral visual regions, perirhinal cortex (PRC), anterolateral EC, and HC. Importantly, these parallel circuits are interconnected on the level of PRC-PHC and EC; and some connections skip levels. Not depicted are differential connectivity patterns between EC subregions and HC subfields, and intrinsic connections within subregions. See “Connectivity and functional implications” Section for details. A, anterior; P, posterior; L, lateral; M, medial.

Anatomical connectivity studies in rodents and nonhuman primates have informed functional accounts of MTL information processing, particularly with respect to memory (Davachi, 2006; Eichenbaum et al., 2007) and, more controversially, perception (Graham et al., 2010) (Note that we focus on visual connections, although all sensory modalities project to the MTL; Burwell, 2000). In humans, evidence mainly comes from functional (e.g., resting-state) connectivity, which often, though not always, aligns with anatomical connectivity (Damoiseaux and Greicius, 2009; Lacy and Stark, 2012). This section outlines how these findings in animals and humans converge (for an overview, see Figure 1).

In animals, MTL [including PRC, PHC, and EC subregions, often denoted lateral and medial EC (LEC, MEC)] is organized in parallel circuits: An anterior circuit connects PRC to LEC, while a posterior circuit connects PHC to MEC (Suzuki and Amaral, 1994a; Burwell and Amaral, 1998a). Both LEC and MEC are in turn connected to hippocampal subfields CA1 and subiculum, albeit distinct parts of these, respectively (Witter and Amaral, 1991; Tamamaki and Nojyo, 1995). Since the MTL gateway regions PRC vs. PHC receive input from ventral vs. dorsal visual regions (Suzuki and Amaral, 1994b; Burwell and Amaral, 1998b), these anterior (PRC, LEC) vs. posterior (PHC, MEC) MTL pathways may process object-related vs. spatial memory representations (Suzuki and Amaral, 1994b; Eichenbaum et al., 2007). However, MTL information flow is more complex: PHC and PRC exhibit both intrinsic connections, and connections with each other (Suzuki and Amaral, 1994b; Lavenex et al., 2004), as do EC subregions (Köhler, 1986, 1988; Dolorfo and Amaral, 1998; Chrobak and Amaral, 2007). LEC and MEC receive additional direct input from regions projecting to PRC and PHC (Burwell and Amaral, 1998b), and some connections between PRC/PHC and HC bypass EC (Suzuki and Amaral, 1990). Note that despite their common designations, LEC vs. MEC may encompass anterolateral vs. posterior-medial, rather than strictly lateral vs. medial EC (Witter et al., 2000).

While fundamental aspects of human MTL functional connectivity—e.g., preferential connectivity of anterior MTL to ventral visual regions, and posterior MTL to dorsal visual regions—may resemble anatomical connectivity in animals (Kahn et al., 2008; Libby et al., 2012), it has been unclear how the fine-scale organization of MTL subregions in animals maps onto human MTL. This concerns the localization of human LEC and MEC homologs as well as their preferential connectivity within and beyond MTL. Two recent, independent studies (Maass et al., 2015; Navarro Schröder et al., 2015) have contributed towards bridging this gap by localizing human EC subfields based on distinct functional connectivity.

Maass et al. (2015) analyzed resting-state functional connectivity *within* MTL using ultra-high field fMRI (0.8 × 0.8 × 0.8 mm resolution at 7 T). EC connectivity gradients emerged along both the anterior-posterior and lateral-medial dimension: PRC vs. PHC showed preferential functional connectivity to antero-lateral EC vs. posterior-medial EC. In turn, these EC subregions showed distinct functional connectivity with proximal and distal, but not anterior and posterior subiculum. Functional connectivity between PRC/PHC and subiculum differed along both the proximal-distal and anterior-posterior subiculum axis. Importantly, these patterns were stable across two independent datasets.

Navarro Schröder et al. (2015), on the other hand, analyzed *large-scale* functional connectivity of EC subregions, again in two datasets: one ultra-high field (0.9 × 0.9 × 0.92 mm resolution at 7 T) task-based fMRI dataset, one resting-state fMRI dataset (2 × 2 × 2 mm resolution at 3 T). Again, differential connectivity emerged along both the anterior-posterior and lateral-medial EC axis. Antero-lateral EC vs. posterior-medial EC showed preferential functional connectivity with the anterior temporal (AT) and posterior-medial (PM) system, two recently proposed large-scale memory systems associated with anterior vs. posterior MTL pathways (Ranganath and Ritchey, 2012).

These results converge on a compartmentalization of human EC into antero-lateral vs. posterior-medial subregions. According to Maass et al. (2015), these may occupy the anterior vs. posterior extremes of EC, and a gradual ratio of lateral vs. medial EC in intervening slices. Functional connectivity of human antero-lateral and posterior-medial EC strongly resembles anatomical connectivity of LEC and MEC observed in animals, suggesting these may be the human homologs of LEC and MEC. Similar functional implications emerge: Antero-lateral vs. posterior-medial EC may preferentially process object-related vs. spatial information relayed to them from ventral and dorsal visual regions via PRC and PHC. The next section therefore discusses functional findings in human EC subregions.

## Material-Specific Processing in Subfields of the Human Entorhinal Cortex

By now, it is well-established that human PRC and PHC—major input regions into the EC—may support encoding (Awipi and Davachi, 2008; Litman et al., 2009; Staresina et al., 2011; Liang et al., 2013) and retrieving (Peters et al., 2007; Staresina et al., 2012, 2013b) object and spatial features, respectively. Rodent LEC and MEC show similar dissociations between object-related and spatial processing (Deshmukh and Knierim, 2011; Yoganarasimha et al., 2011; Hargreaves et al., 2012), in line with their differential connectivity to PRC and PHC outlined above. In humans, however, evidence for material-specific dissociations between EC subfields is relatively sparse, and localization less consistent. Most recently, Navarro Schröder et al. (2015) showed a dissociation for *perceptual* processing. In a high resolution, ultra-high field fMRI dataset, viewing objects vs. scenes was associated with activity in *antero-lateral* vs.* posterior-medial* EC, in accordance with EC subfield localization based on functional connectivity (Maass et al., 2015; Navarro Schröder et al., 2015).

On the other hand, two earlier high-resolution 3T fMRI studies emphasized a *lateral vs. medial* EC distinction, with material-specific differences in *mnemonic* processing (Schultz et al., 2012; Reagh and Yassa, 2014). In Schultz et al. (2012), we examined cue-based retrieval of faces (object-related material) vs. scenes (spatial material) after distraction from working memory. Importantly, this study held perceptual input constant across domains. Reactivation of faces rather than scenes after distraction engaged lateral EC (and PRC), while reactivation of scenes rather than faces after distraction engaged medial EC (and PHC). In Reagh and Yassa (2014), participants encoded object-location associations. During retrieval, ROIs encompassing lateral vs. medial EC halves as well as PRC vs. PHC showed evidence for material-specific interference resolution: They were active during correct rejections of similar objects presented in the same location vs. same objects presented in a different location. In both studies, the lateral-medial distinction appeared dominant: In Schultz et al. (2012), posterior EC showed a lateral-medial pattern qualitatively similar to the main findings, which were present in lateral vs. medial anterior EC. In Reagh and Yassa (2014), a control analysis did not show a significant difference between anterior and posterior EC (although a trend was noted).

**Figure 2:**
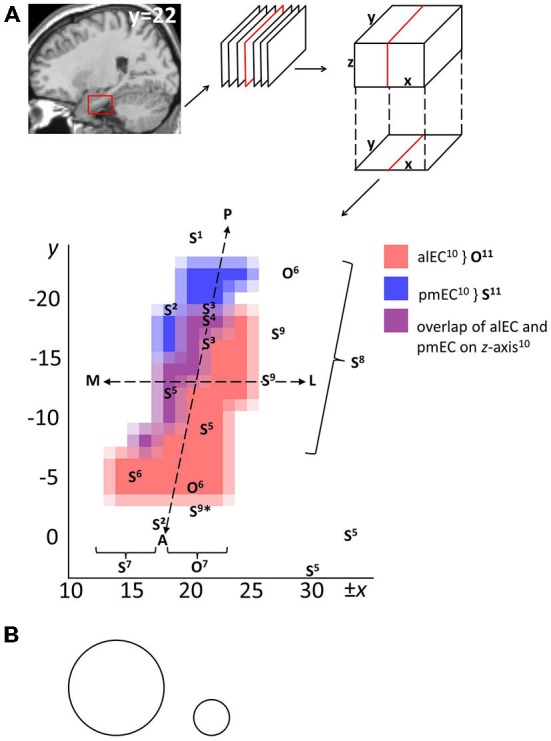
**Overview on human EC subfields based on functional connectivity and localizations of object-related and spatial processing from the literature. (A)** Publically available masks in MNI (Montreal Neurological Institute) space of right antero-lateral and posterior-medial EC (Maass et al., 2015) were projected onto the *x-y* plane and interpolated to a 1 × 1 mm resolution. Alternatively, EC may be approximately divided in lateral and medial halves, or anterior and posterior halves, as demarcated by the superimposed dashed lines. O and S refer to localizations of object-related and spatial functional responses, respectively. Superscript numbers refer to included studies: ^1^Chadwick et al. (2015), ^2^Brown et al. (2014), ^3^Howard et al. (2014), ^4^Spiers and Maguire (2007), ^5^Doeller et al. (2010), ^6^Schultz et al. (2012), ^7^Reagh and Yassa (2014) (functional O vs. S responses in lateral vs. medial regions of interest), ^8^Evensmoen et al. (2015) (change in scale of spatial representations along anterior-posterior axis), ^9^Xu et al. (2010) (*Xu et al., note that their anterior S findings may reflect nonspatial landmark processing), ^10^Maass et al. (2015) (underlying masks), ^11^Navarro Schröder et al. (2015) (functional O vs. S responses in antero-lateral vs. posterior-medial EC, approximately aligning with underlying masks^10^). Note: (1) Peaks were included only if a cluster peaked in EC, multiple peaks from a study were included if they lay in distinct clusters, and some peaks are part of clusters that encompass additional MTL regions such as subiculum and PRC; (2) left-lateralized effects were flipped on the *x*-axis for visualization. Abbreviations: A, anterior; P, posterior; L, lateral; M, medial; alEC, antero-lateral EC; pmEC, posterior-medial EC. **(B)** Example sizes of common Gaussian smoothing kernels, scaled to the dimensions of EC in **(A)**. Circle diameters denote the full width at half maximum value (left: standard resolution fMRI, 8 mm; right: high-resolution fMRI, 3 mm).

Figure 2A relates the above object-related and spatial findings to antero-lateral vs. posterior-medial EC masks provided by Maass et al. (2015). It additionally includes studies that investigated spatial processing without contrasting object-related processing. These implicate EC in route memory (Brown et al., 2014), spatial representations of different granularity (Evensmoen et al., 2015), goal direction (Chadwick et al., 2015), goal distance (Spiers and Maguire, 2007; Howard et al., 2014), computations consistent with grid cell firing (Doeller et al., 2010), and navigational planning (Xu et al., 2010). These spatial peaks appear along the entire anterior-posterior EC axis (Note that (1) Maass et al., 2015 provided functional, not anatomical, EC masks, and (2) that some included studies used standard voxel sizes and smoothing kernels [cf. Figure 2B], potentially contributing to variance in localization).

These findings are not necessarily at odds with the antero-lateral vs. posterior-medial EC distinction (Maass et al., 2015; Navarro Schröder et al., 2015). In studies contrasting object-related and spatial processing, differences mainly pertain to the relative dominance of the anterior-posterior (Navarro Schröder et al., 2015) or lateral-medial dimension (Schultz et al., 2012; Reagh and Yassa, 2014). Nevertheless, a majority of coronal EC slices contain both antero-lateral EC and posterior-medial EC (Maass et al., 2015). Navarro Schröder et al. (2015) also observed lateral-medial effects for object vs. scene viewing in some coronal slices containing both subregions, and argued that earlier findings of lateral-medial dissociations are largely consistent with an antero-lateral vs. posterior-medial distinction. Spatial processing along much of the anterior-posterior EC axis would similarly fit in this framework (see Figure 2A).

However, we suggest that additional, largely processual, factors may further modulate localization of material-specific effects in EC. As pointed out above, the studies reporting material-specific effects primarily for lateral vs. medial EC investigated *memory retrieval* (Schultz et al., 2012; Reagh and Yassa, 2014), while Navarro Schröder et al. (2015) investigated *perceptual* processes. During perception and encoding, material-specific information may travel from ventral and dorsal visual pathways via PRC/PHC and EC to the HC, while retrieval signals may take the opposite route (Naya et al., 2001; Eichenbaum et al., 2007; Staresina et al., 2013b). Furthermore, spatial processing in EC may be partly computed by grid cells—cells in rat (dorsocaudal) MEC that fire in a grid-like pattern depending on an animal’s position in space (Fyhn et al., 2004; Hafting et al., 2005). Their existence in the human brain is supported directly and indirectly through fMRI (Doeller et al., 2010), cell recordings (Jacobs et al., 2013), and behavioral studies (Chen et al., 2015). In primates, visual scene exploration, rather than actual spatial exploration of the environment, may suffice to elicit activity in grid cell ensembles, particularly in posterior EC (Killian et al., 2012). Therefore, grid cell ensembles may be more engaged during scene viewing than retrieval. Furthermore, the scale of spatial representations may change along the anterior-posterior EC axis (Evensmoen et al., 2015), which may further modulate the localization of spatial processing. Demand for information integration may also influence localization of functional EC subregions: integration of object-related with spatial (memory) representations may be supported by intrinsic anatomical EC connectivity (Lavenex and Amaral, 2000; Knierim et al., 2014) (see also the following section), and Navarro Schröder et al. (2015) reported an additional functional EC connectivity gradient, possibly reflecting differences in intrinsic (rather than extrinsic, see “Connectivity and functional implications” Section) connectivity. Speculatively, human EC subregions may support differential, or integrated, processing of both material types, rather than specialization for one material: e.g., in Schultz et al. (2012), the face distraction effect in lateral EC was reversed for scenes, and the Reagh and Yassa (2014) task required retrieving object-location associations while resolving object or spatial interference. Finally, an earlier fMRI study did not investigate EC subfields, but located object-related vs. spatial processing to *left* vs. *right* EC, (Bellgowan et al., 2009). Likewise, Doeller et al. (2010) noted that effects of spatial processing may be more reliable in right EC. This suggests potential laterality effects.

## Outlook: Information Integration in the Entorhinal Cortex

Recently observed dissociations between object-related vs. spatial processing in human EC subregions are not unique to the EC: material-specific retrieval in lateral vs. medial EC (Schultz et al., 2012; Reagh and Yassa, 2014) closely resembles effects in PRC vs. PHC in these same studies. Navarro Schröder et al. (2015) reported effects of object vs. scene viewing in antero-lateral vs. posterior-medial EC. Previous studies observed similar dissociations in PRC vs. PHC (Litman et al., 2009; Liang et al., 2013). These findings support a model in which EC serves as a relay station between PRC/PHC and the HC, though EC may be more than that (Kerr et al., 2007; van Strien et al., 2009; Knierim et al., 2014). This raises the question of what computations the EC may uniquely contribute to the processing of object-related and spatial memory representations. Again, insight may be gained from animal studies on connectivity and function.

Information represented in EC likely does not overlap completely with that in PRC/PHC: In animals, some projections to EC from ventral and dorsal visual regions are direct rather than relayed through PRC/PHC, and in turn some projections from PRC/PHC to HC bypass EC (Suzuki and Amaral, 1990; Burwell and Amaral, 1998b).

Additionally, a high degree of intrinsic EC connectivity across subfield boundaries (Köhler, 1986, 1988; Dolorfo and Amaral, 1998; Chrobak and Amaral, 2007) implies that object-related and spatial information is integrated to a certain degree (Lavenex and Amaral, 2000; Knierim et al., 2014). Indeed, studies in rats suggest that unlike PRC (Deshmukh et al., 2012), LEC may integrate object-related representations with spatial, or contextual information (Deshmukh and Knierim, 2011; Hunsaker et al., 2013; Tsao et al., 2013; Van Cauter et al., 2013; Wilson et al., 2013). In turn, MEC may also process nonspatial information (Hunsaker et al., 2013).

Spatial activity in MEC, unlike PHC, may be partially supported by grid cell activity, as discussed above. EC grid cells may be part of both navigation and memory circuits (Sasaki et al., 2015). Thus, MEC and PHC may not only make differential contributions to spatial navigation, but to (spatial) memory in general. Attempts to integrate the spatial navigation and memory functions of MTL have been made elsewhere (Buzsáki and Moser, 2013; Maguire and Mullally, 2013; Eichenbaum and Cohen, 2014; Buffalo, 2015).

In sum, material-specific representations in both rodent LEC and MEC may differ from those in PRC and PHC. On these grounds, it has been argued that rodent LEC vs. MEC process content vs. (spatial) context, with LEC computing object-related and spatial processing within a local reference frame and MEC providing a global spatial reference frame (Neunuebel et al., 2013; Knierim et al., 2014). Future research may reveal what aspects of the object-related vs. spatial processing observed in human EC may reflect information relay between PRC/PHC and HC, and what aspects may be computations specific to EC.

Finally, while we focused on different memory representations based on visual input, EC receives more than just object-related and spatial information. As just one example, regions implicated in reward and emotion project directly to EC (Beckstead, 1978; Insausti et al., 1987; Oades and Halliday, 1987). Thus, highly associative EC memory representations may also integrate some of their motivational properties. While this has not been investigated in humans, primate EC may play a role in reward-related learning (Sugase-Miyamoto and Richmond, 2007). Additionally, antero-lateral (vs. posterior-medial) EC may be connected to the AT (vs. PM) system (Navarro Schröder et al., 2015), as proposed by Ranganath and Ritchey (2012), including PRC, amygdala, lateral orbitofrontal and ventral temporopolar cortex. Thus, specifically antero-lateral EC may be part of a system proposed to represent motivational aspects of memory. A thorough account of memory representations in human EC therefore has to consider its wide-spread connectivity with other limbic and neocortical circuits.

## Conclusion

Recent evidence suggests that human EC may be divided into antero-lateral vs. posterior-medial subfields with object-related vs. spatial preferences, in line with the animal literature. This reinforces a representational MTL model of memory, in which the HC receives object-related information from ventral visual areas via PRC and (antero-lateral) EC, and spatial information from dorsal visual areas via PHC and (posterior-medial) EC (Eichenbaum et al., 2007). Within this model, EC may act as more than a relay station, as implied by its specific properties including strong intrinsic connectivity between subfields, and unique computational properties (e.g., grid cells). Future research may characterize specific contributions of human EC subregions to object-related and spatial processing as distinguished from PRC and PHC, and consider EC’s widespread cortical and subcortical connectivity to regions outside the MTL. Given that EC is one of the earliest regions affected in AD, a thorough characterization of its function may have important clinical implications.

## Conflict of Interest Statement

The authors declare that the research was conducted in the absence of any commercial or financial relationships that could be construed as a potential conflict of interest. The Review Editor Kelly Giovanello declares that, despite being affiliated with the same institution as the Associate Editor Charlotte Boettiger, the review process was handled objectively.
